# *De novo* design of mIDH1 inhibitors by integrating deep learning and molecular modeling

**DOI:** 10.3389/fphar.2024.1491699

**Published:** 2024-10-23

**Authors:** Dingkang Sun, Lulu Xu, Mengfan Tong, Zhao Wei, Weitong Zhang, Jialong Liang, Xueying Liu, Yuwei Wang

**Affiliations:** ^1^ College of Pharmacy, Shaanxi University of Chinese Medicine, Xianyang, China; ^2^ Thyroid and Breast Surgery, General Hospital of Xinjiang Military Comand, Xinjiang, China; ^3^ State Key Laboratory of Holistic Integrative Management of Gastrointestinal Cancers and National Clinical Research Center for Digestive Diseases, Xijing Hospital of Digestive Diseases, Fourth Military Medical University, Xi’an, China; ^4^ Department of Medicinal Chemistry, School of Pharmacy, Air Force Medical University, Xi’an, China; ^5^ No. 946 Hospital, Xinjiang Uygur Autonomous Regions, Xinjiang, China

**Keywords:** mIDH1, BRNN model, scaffold hopping, virtual screening, molecular dynamics

## Abstract

**Background:**

Mutations in the IDH1 gene have been shown to be an important driver in the development of acute myeloid leukemia, gliomas and certain solid tumors, which is a promising target for cancer therapy.

**Methods:**

Bidirectional recurrent neural network (BRNN) and scaffold hopping methods were used to generate new compounds, which were evaluated by principal components analysis, quantitative estimate of drug-likeness, synthetic accessibility analysis and molecular docking. ADME prediction, molecular docking and molecular dynamics simulations were used to screen candidate compounds and assess their binding affinity and binding stability with mutant IDH1 (mIDH1).

**Results:**

BRNN and scaffold hopping methods generated 3890 and 3680 new compounds, respectively. The molecules generated by the BRNN performed better in terms of molecular diversity, druggability, synthetic accessibility and docking score. From the 3890 compounds generated by the BRNN model, 10 structurally diverse drug candidates with great docking score were preserved. Molecular dynamics simulations showed that the RMSD of the four systems, M1, M2, M3 and M6, remained stable, with local flexibility and compactness similar to the positive drug. The binding free energy results indicated that compound M1 exhibited the best binding properties in all energy aspects and was the best candidate molecule among the 10 compounds.

**Conclusion:**

In present study, compounds M1, M2, M3 and M6 generated by BRNN exhibited optimal binding properties. This study is the first attempt to use deep learning to design mIDH1 inhibitors, which provides theoretical guidance for the design of mIDH1 inhibitors.

## 1 Introduction

The tricarboxylic acid cycle (TCA cycle) is an important metabolic pathway in aerobic organisms (Scagliola et al., 2020). Isocitrate dehydrogenase (IDH) is an important rate-limiting enzyme in this pathway, which catalysis the oxidative decarboxylation of isocitrate to produce α-ketoglutarate (α-KG) and CO_2_ (Dang et al., 2016). The IDH family consists of three isozymes: IDH1 in the cytoplasm, and IDH2 and IDH3 in the mitochondria (Tian et al., 2022). Among them, IDH1 plays important functions in cellular energy supply, redox balance, biosynthesis and signal transduction, etc. (Waitkus et al., 2018). Small variations in its structure and function can trigger a chain reaction and promote the formation and development of tumors. IDH1 mutations occur mainly in the early stage of tumors, especially in gliomas and glioblastoma multiforme. IDH1 mutations include five types: R132H/C/L/S/G, of which R132H mutations are the most common, accounting for 30% of all mutations (Platten et al., 2021).2-Hydroxyglutaric acid (2-HG) has a relatively low content at normal physiological levels, but IDH1 mutations lead to abnormally high levels of 2-HG (Yan et al., 2009). 2-HG is structurally similar to α-KG and can competitively inhibit α-KG and occupy the active site of α-KG-dependent dioxygenase. This inhibition hinders the conversion of 5-methylcytosine to 5-hydroxycytosine, thereby impairing the process of DNA demethylation. At the same time, it inhibits the expression and regulation of histone demethylases of the JmjC structural domain during cell differentiation, leading to DNA hypermethylation and epigenetic dysregulation. This greatly disrupts the normal physiological activities of the organism (Golub et al., 2019).

Currently there are many investigational mIDH1 inhibitors in the clinic. For example, Mindy et al. first reported the mIDH1 inhibitor ML309 with phenylglycine backbone in 2012, and ML309 showed good selectivity between wild-type and R132H mutant IDH1, which could effectively reduce the production of 2-HG in the U87MG cell line (Davis et al., 2014). Dan et al. optimized the structure of ML309 to obtain the compound AGI-5198, which has an IC_50_ value of 70 nM for inhibiting the activity of IDH1 R132H enzyme. However, its metabolism and clearance *in vivo* and its poor druggability have limited its further clinical application (Rohle et al., 2013; Popovici-Muller et al., 2012). Based on the molecular structure of AGI-5198, Angios designed and synthesized Ivosidenib, which is highly selective for IDH1 mutants. Ivosidenib was also the first mIDH1 inhibitor to be approved for clinical use by the FDA, but has been reported to develop resistance during treatment. Despite the current progress in research on IDH1-type mutant inhibitors, there is still a lack of effective mIDH1 inhibitors in the clinic. Therefore, the development of novel, highly selective small molecule inhibitors targeting IDH1 mutants is important to improve the efficacy of tumor therapy and reduce drug resistance.

The search for new drugs is a long, expensive and difficult process. Studies have shown that the number of chemically synthesizable active compounds is estimated to be 10^30^ to 10^60^, which is a huge amount of work and inefficient for traditional drug discovery based high throughput screening (Polishchuk et al., 2013; Macarron et al., 2011). Whereas designing molecules with desired properties from scratch can face complex multivariate optimization tasks (Macarron et al., 2011). Computational methods have proved to be valuable in generating new molecules (Munk, 1998). Studies have shown that generative deep learning techniques, such as Recurrent Neural Networks (RNN), have emerged as a potential alternative to rule-based methods for designing molecules from scratch (Rumelhart et al., 1985; Hopfield, 1982). RNN can use SMILES strings to generate new chemical molecular structures. Typically, RNN-based methods operate unidirectionally in generating molecular structures, i.e., constructing SMILES strings step by step in a left-to-right order (Gómez-Bombarelli et al., 2018). However, considering that small molecules themselves do not have a fixed start or end point, and that SMILES strings are a non-unique representation of molecular maps, this suggests that we could try a bidirectional approach to generating molecular structures. This means that not only can the SMILES string be grown from left to right during the generation of molecules, but also right-to-left or simultaneous bi-directionality can be considered as a way to increase the efficiency and diversity of the generation (Grisoni et al., 2018). Francesca Grisoni and Gisbert Schneider et al. constructed a bi-directional strategy for molecular design from scratch (BRNN) based on SMILES, and by comparing the novelty, backbone diversity, and chemical-biological relevance of the molecules generated by uni-directional RNN versus BRNN, it was found that most of the standard BRNN showed superior properties than the uni-directional RNN for most of the standard BRNN under the tested conditions (Grisoni et al., 2020). Therefore, the BRNN model was used in this study for the design of mIDH1 inhibitors. Scaffold hopping are for the functional groups of compounds for fragment substitution based on the principle of electron isomers (Sun et al., 2012), whereas the BRNN model is an encoder-decoder architecture based on the attention mechanism for the prediction of fragments (Polishchuk et al., 2013; Hopfield, 1982). Specifically, the encoder of the BRNN model consists of a bi-directional RNN that processes the input vectors and then sums the resulting hidden states in both directions into the decoder. The bi-directional RNN consists of a forward RNN and a backward RNN, making full use of string future and past features. The decoder is a unidirectional RNN that predicts the next character by decoding the previous state vector. Then the global attention mechanism is applied to give different weights and attention to the outputs of the information in the hidden layer to further learn the intrinsic correlation information of the skeleton and fragment structure (Grisoni et al., 2020). In this way, the BRNN model is able to learn and understand the intrinsic connection between the compound backbone and its fragment structure in a deeper way, leading to more accurate fragment prediction.

**FIGURE 1:**
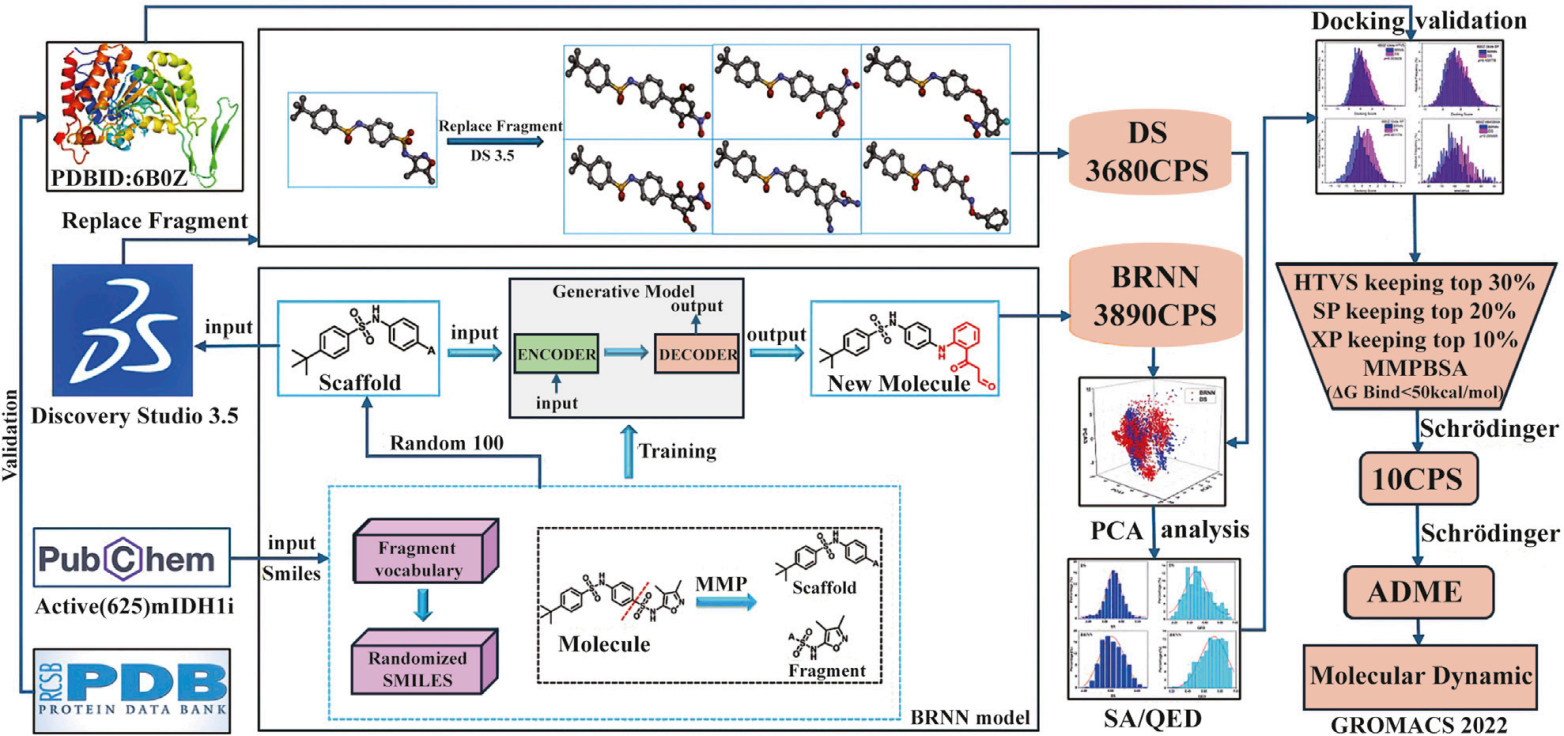
Workflow for mIDH1 inhibitor design based on BRNN model, DS Scaffold Hopping, molecular docking and molecular dynamics simulations.

In this study, the BRNN model and scaffold hopping were used to generate mIDH1 inhibitors. Based on the superiority of the molecules generated by the two methods, the molecules generated by the BRNN model were selected for virtual screening, and the optimal molecules were subjected to molecular dynamics simulations. The specific workflow is shown in Figure 1.

## 2 Materials and methods

### 2.1 Experimental environment

The computing platform used in this study consists of a Bauder PR271ORN 2U rackmount server with an Intel Xeon GOLD 6248R CPU and an NVIDIA V100S graphics card. The software environment includes Discovery Studio 3.5, Schrödinger 2020 and GROMACS 2022.

### 2.2 BRNN models

**FIGURE 2:**
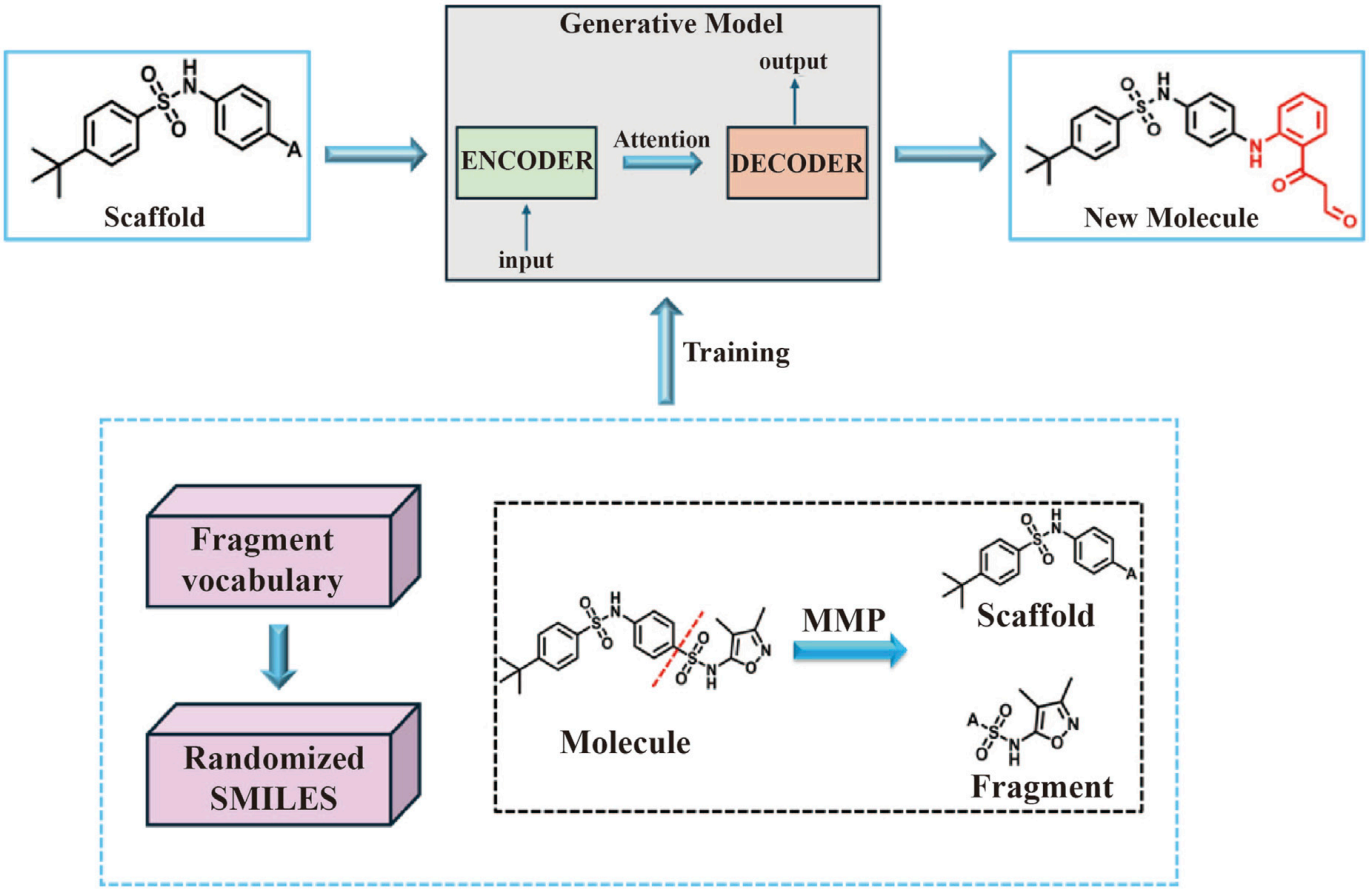
Workflow for generation of mIDH1 inhibitors based on the BRNN model.

In this study, novel small molecules with potential mIDH1 inhibitory activity were generated using BRNN models (https://github.com/ETHmodlab/BIMODAL) based on TensorFlow (Grisoni et al., 2020). The methodology is as follows: first, the smiles format of 625 active compounds were downloaded from the PubChem(https://pubchem.ncbi.nlm.nih.gov) database for feature learning. Second, the MMP algorithm proposed by Arús-Pous was used to split the 625 mIDH1 inhibitors (Arús-Pous et al., 2020), which obtains a tuple of skeleton-decorated fragments by cutting all possible combinations of acyclic bonds in each molecule. In order to distinguish backbone from decorated fragments, the following restrictions were added to the MMP algorithm: backbone fragments need to contain at least one ring; decorated fragments need to satisfy certain criteria (Congreve et al., 2003), i.e., HBD ≤5, ClogP ≤5, and RotBonds ≤5. A library of backbone-decorated fragments containing 27,711 molecule pairs was finally obtained, which was divided into training set, testing set and validation set in the ratio of 8:1:1, and the generative model was trained to learn potential information about how the skeleton molecules are connected to the decorated fragments. While the model was trained and parameters optimally tuned using the training set and validation set, the valid accuracy of the model was analyzed using the test set. In order to fully understand the connectivity features of the different structures of the mIDH1 inhibitor, a molecularly generated model containing four hidden layers as well as 1,272,967 tunable parameters was constructed using BRNN. After model training, simulated sampling was performed using the optimized model for a total of 10,000 samples. The sampled molecules were used to compute the InChI descriptors of the molecules using the RDInChI module in the RDKit (https://github.com/rdkit) package and the duplicate molecules were removed. The obtained molecules were stored in SDF format and the molecules with MW > 500 was filtered out (Figure 2).

### 2.3 Scaffold hopping

The Lead Optimization module of DS 3.5 software (Sheoran et al., 2023)was used to carry out molecular scaffold hopping. Randomly select 100 IDH1 inhibitors split by the MMP algorithm was imported into the DS 3.5 software, and then select the Fragment part of the molecule and choose the Replace Fragment module to replace the Fragment part of these 100 IDH1 inhibitors *in situ*, and the threshold for the number of molecules to be generated to 100.

### 2.4 SA, QED and PCA analysis

In the development of new drug candidates, it is important to consider their synthetic accessibility (SA) and drug-like properties. In this study, chemical synthesizability was assessed using SA based on fragment contribution and complexity penalty. This approach defines SA as a score between 1 (easy to synthesize) and 10 (difficult to synthesize) (Ertl et al., 2000). The assessment of drug-like properties was based on the quantitative estimated drug (QED) similarity index, which measures how closely a given compound resembles currently known drugs in terms of structural and physicochemical properties (Roy and Kadam, 2007). The index value ranges from 0 to 1, with closer to 1 indicating better drug-like properties. Then, the SA and QED scores of the new molecules generated by the DS 3.5 and BRNN models were calculated separately through RDKit package. In order to quantify the chemical properties of small molecules, the rdkit. Chem.Descriptors.descList function of the RDKit package was employed to generate approximately 200 different molecular descriptors, which encompass a wide range of molecular properties, including basic molecular weight and LogP, as well as more complex topological and geometric descriptors, thereby providing a comprehensive description of the physical, chemical, and structural properties of molecules. Furthermore, the PCA method was utilized to decrease the data’s dimensionality and aid in visual analysis.

### 2.5 Redocking validation

The co-crystal ligands of the 6B0Z protein were redocked using Schrödinger 2022 (Onikanni et al., 2023). The validation of the docking results was based on the RMSD (Root Mean Square Deviation) value (indicating the degree of deviation), i.e., the higher the RMSD value, the higher the deviation. If the RMSD value is less than 2 Å, the method is dependable (Khan et al., 2022). The BRNN model and the molecules generated by DS 3.5 were docked using three docking modes, HTVS, SP and XP, respectively, in the Glide module of Schrödinger software.

### 2.6 Processing of crystal structures

In the previous study, nine crystal structures of mIDH1 (PDB ID: 5LGE, 5SUN, 5SVF, 5TQH, 4UMX, 5L57, 5L58, 6ADG, 6B0Z) were obtained from the PDB database, and the docking and enrichment abilities of each crystal structure were evaluated using cross-docking (Wang et al., 2020). Ultimately, it was found that targeting the 6B0Z conformation with the best enrichment ability could better screen mIDH1 inhibitors accurately from the virtual compound database. After the 6B0Z crystal structure was imported into Schrödinger 2022, Protein Preparation Wizard module was used to pre-process the crystal structure, including the removal of water molecules, hydrogenation, charging, creation of disulfide bonds and complementary residues. Restriction optimization of the protein backbone was then performed using the OPLS_2005 force field (Tiwari et al., 2018). The Receptor Grid Generation module was then used to generate hexahedral boxes of similar size to the native ligand (IDH305) as the center of the grid in the protein structure defined as the binding pocket. IDH305 demonstrates an IC_50_ value of 18 nM against the IDH1 R132H enzyme, exhibiting approximately 200-fold greater selectivity compared to the wild-type IDH1 enzyme (Cao et al., 2019). Preclinical studies have shown that IDH305 effectively reduces the level of 2-HG in tumors. Due to its favorable pharmacological activity and pharmacokinetic properties, IDH305 was employed as a positive control in the study (Cho et al., 2017).

### 2.7 Preparation of ligand

Molecular libraries generated through the BRNN model and DS scaffold hopping were used as a screening database, containing approximately 7,570 molecules. Small molecule processing was performed using the LigPrep module of the Schrödinger 2022 with the OPLS-2005 force field, and Epik module was used to generate the possible states of the molecules at pH 7.0 ± 2.0, followed by the generation of tautomer, with up to 10 low-energy conformations per molecule (Shelley et al., 2007).

### 2.8 ADME prediction and prime MM-GBSA

The ADME (Absorption, Distribution, Metabolism and Excretion) properties of the compounds are determined using the QikProp tool of the Schrödinger 2022 software. The QikProp tool calculates physically meaningful descriptors and pharmaceutically relevant properties of organic molecules, enabling rapid and accurate ADME prediction and the early identification of problematic small molecule candidates, significantly reducing time and resources (Patel H. M. et al., 2020). Drug similarity and drug factors were evaluated for small molecules with all hits (Daina et al., 2017). In Maestro, the binding free energy of potential inhibitors to the mIDH1 crystal structure was calculated using the Prime MM-GBSA method for assessing the strength of interaction between the ligand and the target protein (Bouzian et al., 2024).

### 2.9 Glide-based virtual screening

The generated grid and the prepared ligands were underwent virtual screening using Glide’s virtual screening workflow (Friesner et al., 2006). The prepared ligands were prefiltered with QikProp to ensure they had the necessary properties (Lipinski et al., 1997). The initial stage involved HTVS docking for rapid high-throughput screening. The top 30% of molecules from this stage moved on to SP docking, which retains the top 20% of molecules. Molecules that passed this selection process proceeded to the final stage, where XP docking was performed and only the top 10% of molecules were retained.

### 2.10 Molecular dynamics simulations

Molecular dynamics simulations for the conformations with the outcomes of docking for candidate small molecules were conducted using the GROMACS 2022 with CHARMM 36 force field. Prior to simulation, all molecules underwent hydrogen addition and deprotonation processes executed through the Avogadro software. Subsequently, the CgenFF (Bouchouireb et al., 2024) (https://cgenff.silcsbio.com, accessed on 9 July 2024) was employed to generate parameters of small molecules. The complexes were then solvated in a cubic box of TIP3P water, maintaining a distance of 10 Å from the protein, with the box thickness ensuring a minimum of 1 nm clearance. To neutralize the system, sodium and chloride ions were introduced, while periodic boundary conditions (PBCs) were imposed in all dimensions to mitigate edge effects during the simulation. Relevant long-range electrostatic interactions were calculated using the Particle-Mesh-Ewald (PME) method. Following optimization, the ensemble was subjected to 100ps of NVT and NPT equilibration phases at 300 K and 1 atm, respectively. The actual molecular dynamics simulations were carried out for 400 ns with a time step of 2 fs. Post-simulation analyses comprised the root mean square deviation (RMSD) of each complex and the root mean square fluctuation (RMSF) of residues within the protein, providing insights into structural stability and flexibility.

### 2.11 Calculation of binding free energy

The MM-PBSA method offers a straightforward way to measure the free energy of binding between receptors and ligands (Miller et al., 2012). In this study, the binding affinities of simulated receptor-ligand complexes were calculated using the gmxapbs tool in GROMACS, employing specific formulas as outlined below (Kumari et al., 2014):

$${\Delta G}_{bind}={\Delta G}_{complex}-\left({\Delta G}_{protein}+{\Delta G}_{ligand}\right)$$

$${\Delta G}_{bind}={\Delta E}_{MM}+{\Delta E}_{PB}+{\Delta E}_{SA}-T\Delta S$$

$$\Delta G={\Delta G}_{bind}=\Delta H-T\Delta S$$

$${\Delta E}_{binding}={\Delta H=\Delta E}_{MM}+{\Delta E}_{PB}+{\Delta E}_{SA}$$

$${\Delta E}_{MM}={\Delta E}_{COU}+{\Delta E}_{VDW}$$

$${\Delta E}_{PB}={\Delta E}_{PBcom}-\left({\Delta E}_{PBpro}+{\Delta E}_{PBlig}\right)$$

$${\Delta E}_{SA}={\Delta E}_{SAcom}-\left({\Delta E}_{SApro}+{\Delta E}_{SAlig}\right)$$

In brief, a total of 5,000 snap-shots were extracted from the stable simulation trajectories of each system over the last 10 ns, with a 2 ps interval, for MM/PBSA calculation.

### 2.12 Computation of DCCM and FEL

In this study, the covariance matrix was calculated using the Covar command of GROMACS. The covariance matrix data were then converted into dynamic correlation maps (DCCM) (https://github.com/busrasavas). The free energy landscape (FEL) was plotted using RMSD and Gyrate, and the trajectories were plotted using simulated post-stabilization protein trajectories at 20 ns and 3D free energy landscape maps.

## 3 Result

### 3.1 Performance of BRNN models

**FIGURE 3:**
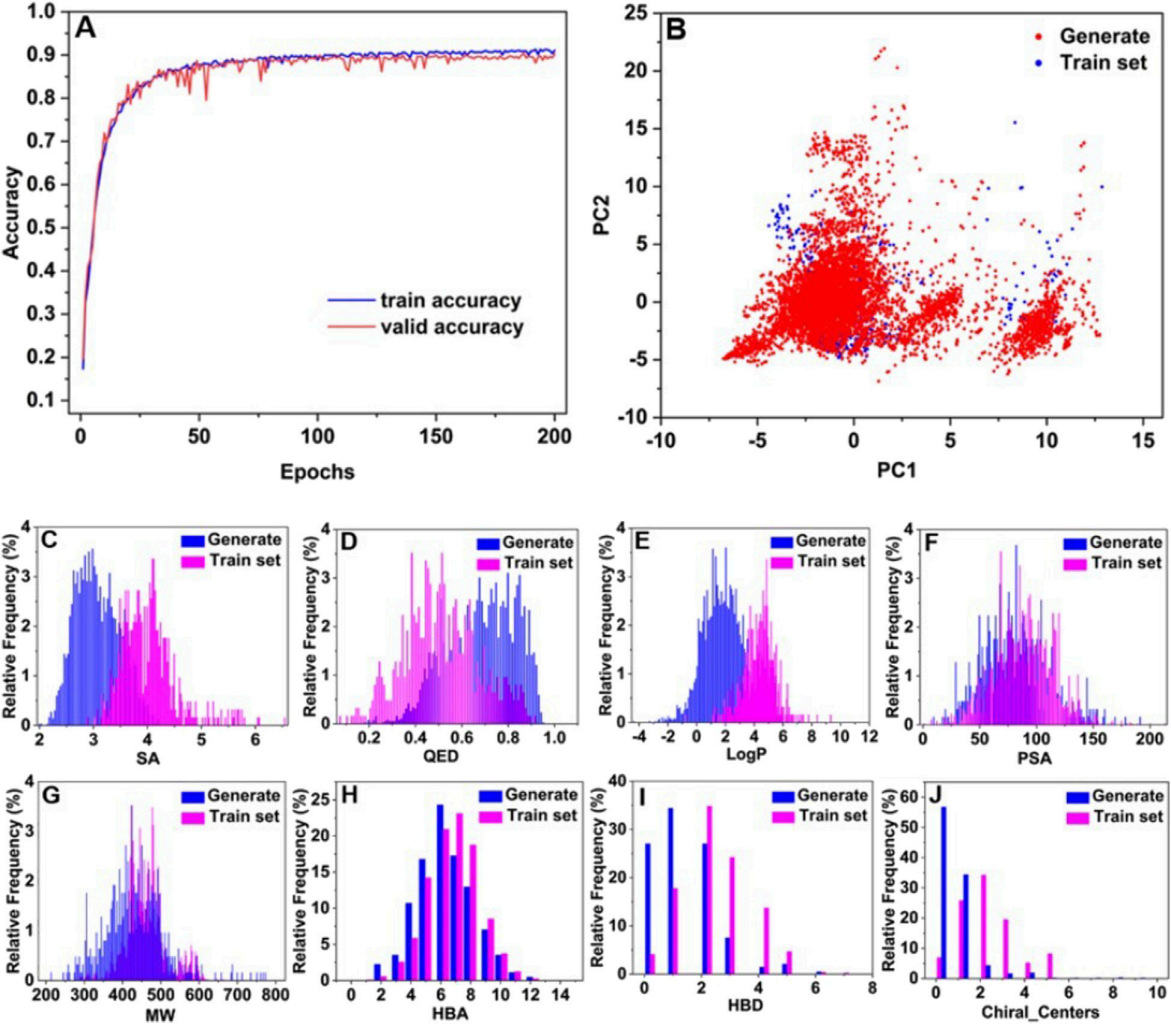
Plot of BRNN model training accuracy over time **(A)**, and chemical space analysis and distribution of physicochemical properties **(B–J)**.

The model was trained for 200 Epochs using the Adam optimizer, and the generated model converged after 75 Epochs, and the loss value did not decrease further after 100 Epochs (Figure 3A). The model generated by the 100th Epoch was used as the subsequent decorated model to build a library of skeleton-based molecules by modifying the skeletons of 100 randomly selected split mIDH1 inhibitors. A total of 9,736 new molecules were generated, which were decorated and screened for new molecules with MW > 500, resulting in 3,890 new compounds.

To visualize the distribution of the generated molecules in the chemical space relative to each other, PCA was used for dimensionality reduction to map the high-dimensional data into a two-dimensional representation. Figure 3B displays the diversity distribution maps of the PCA chemical space for the molecules generated by the BRNN model (Generate) and the 625 mIDH1 inhibitors in 2.2 (Train set). The results of PCA analysis showed that the molecules generated by BRNN and Train set molecules were mainly divided into two clusters in terms of chemical space distribution, with the left clusters accounting for the majority and the right clusters accounting for a small portion. The molecules generated by BRNN in the two clusters cover a larger area and a wider chemical space, almost completely covering the chemical space of the Train set molecules and filling in part of the empty space around them, which indicates that the molecules generated by BRNN are more diversified, and also reflects that the BRNN model is not only capable of generating structures similar to those of the active molecules but also of generating completely new molecules. In order to evaluate the quality of the generated molecules, the physicochemical properties such as synthesizability, drug-like properties (QED), and water-octanol partition coefficient (LogP) of the generated molecules and the train set molecules were calculated separately. Figures 3C–J demonstrate the distribution of physicochemical properties of the generated molecule library versus the train set molecules. It can be seen from Figure 3C that the highest point of the distribution of SA scores of the generated molecules is at 2.9, while the highest point of the distribution of SA scores of the train set molecules is at 4. The SA scores of the generated molecules are more skewed towards 1, showing a left-skewed distribution, which suggests that the generated molecules have better synthesizability. The highest point in the distribution of QED scores of the train set molecules in Figure 3D was at 0.5 and the overall distribution was skewed towards 0, while the highest point in the distribution of QED scores of the generated molecules was at 0.65 and the overall distribution was skewed towards 1, suggesting that the generated molecules had a more higher drug-like properties. The distribution of LogP values of the newly generated molecules is closer to −4 relative to the train set molecules (Figure 3E), indicating that the newly generated molecules have better water solubility and are more readily absorbed through the gastrointestinal mucosa, resulting in better oral bioavailability. Based on the characteristics of extended rule of five (eRo5):MW ≤ 500, HBD ≤ 5, HBA ≤ 10, LogP ≤ 5, PSA ≤ 200 (Doak et al., 2014), it was observed that the distribution of PSA (Figure 3F), MW (Figure 3G), HBA (Figure 3H) and HBD (Figure 3I) of newly generated molecules shifted towards lower values compared to those in the train set. This observation suggests that the newly generated molecules exhibit a more drug-like profile.

Chiral_Centers indicates the number of chiral centers of a compound, and too many chiral centers can lead to a significant increase in the difficulty of the synthesis and purification process (Li et al., 2024). As shown in Figure 3J, the number of chiral centers of the generated molecules is distributed below 2 for about 90% of the generated molecules, while the train set molecules are distributed below 2 for about 60% of the generated molecules. The number of chiral centers of the generated molecules is lower than that of the train set molecules, which further explains that the generated molecules have better synthesizability than the Train set molecules. To further evaluate the diversity of the generated molecules, this study applied RDKit to calculate the Bemism-Murcko skeleton of each molecule generated, which totaled 3,890 molecules with a total of 1,789 Bemism-Murcko skeletons. This analysis also confirms the feasibility of the generative model to generate a diverse library of molecules based on backbones.

### 3.2 Generation of mIDH1 inhibitors based on scaffold hopping

Each of the 100 scaffolds produced 90–400 new molecules after hopping, resulting in a total of 18,770 new molecules. The compounds with molecular weight less than 500 were screened and ranked by molecular similarity. Compounds with fragment similarity greater than 0.6 were screened, and a total of 3,680 new compounds were obtained.

### 3.3 Chemical spatial analysis of molecules generated by BRNN and scaffold hopping

**FIGURE 4:**
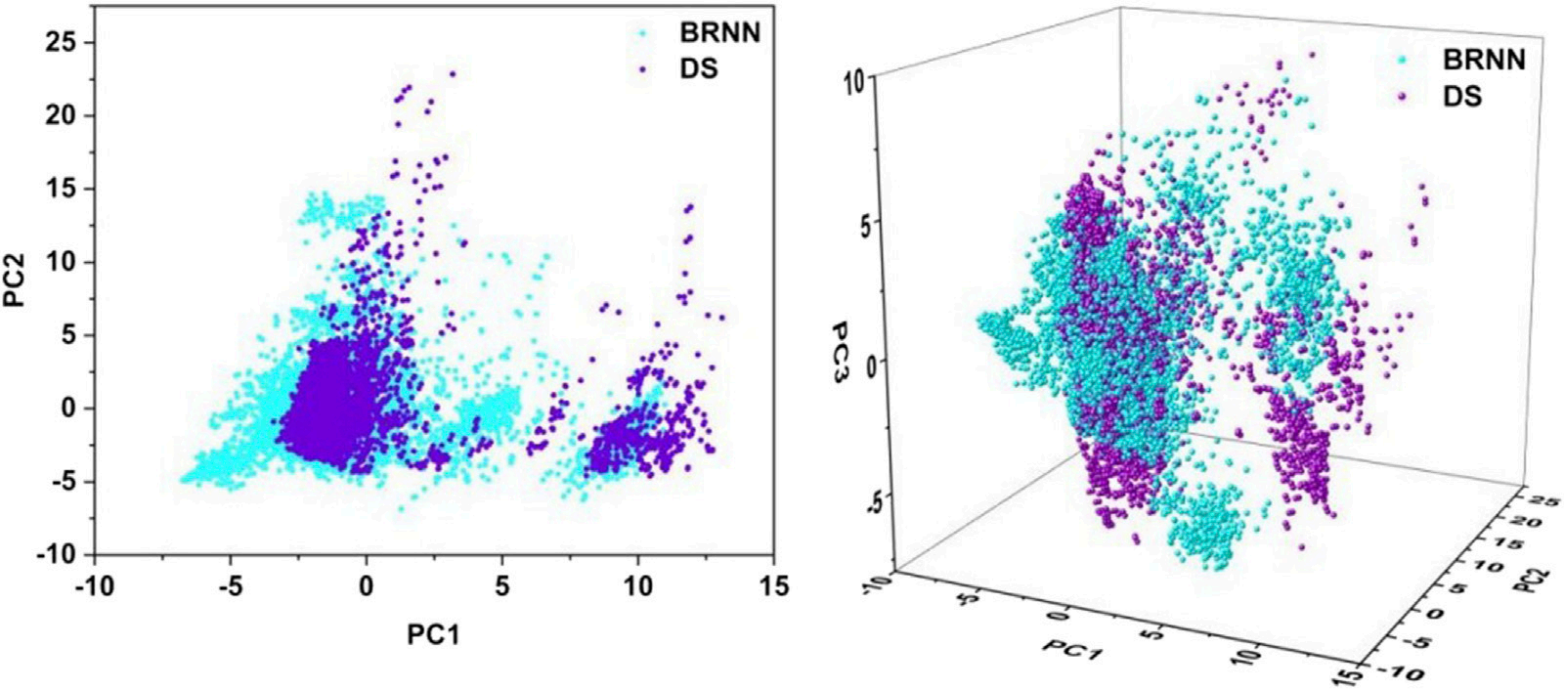
Principal component analysis of molecules generated by BRNN and scaffold hopping.

In order to reduce the dimensionality of the data and facilitate visual analysis, the principal component analysis (PCA) method was employed. The application of PCA allows the reduced-dimensional representation of the BRNN model and DS framework to be visualized in two or three dimensions (Figure 4). The 2D PCA representation employs two principal components to illustrate the distribution of data in a two-dimensional space, which facilitates the identification of similarities and differences between compounds, particularly in the context of key molecular descriptors. Conversely, 3D PCA provides a more comprehensive spatial perspective, enabling further differentiation of these distinctions. As illustrated in the Figure 4, the 2D plot shows that the results of the PCA analysis are mainly divided into two clusters, with the left cluster accounting for the majority and the right cluster accounting for a small portion. The molecules generated by BRNN in the left cluster cover a larger area and have a wider chemical space, indicating that the molecular diversity generated by BRNN in this cluster is higher. The molecules generated by DS in the right cluster cover a larger area, indicating a higher diversity of DS-generated molecules in this cluster. The molecular distributions generated by the BRNN model and the DS-generated molecules show some overlap, as well as a clear clustering pattern. This distribution model suggests that the two groups of compounds exhibit some similarity in chemical and physical properties. The 3D plots show that the coverage of BRNN-generated molecules and DS-generated molecules is more similar, with BRNN molecules appearing wherever DS molecules appear. Overall, BRNN-generated molecules cover a larger area, indicating that BRNN-generated molecules are more diverse than DS-generated molecules.

### 3.4 Distribution of QED and SA of molecules

**FIGURE 5:**
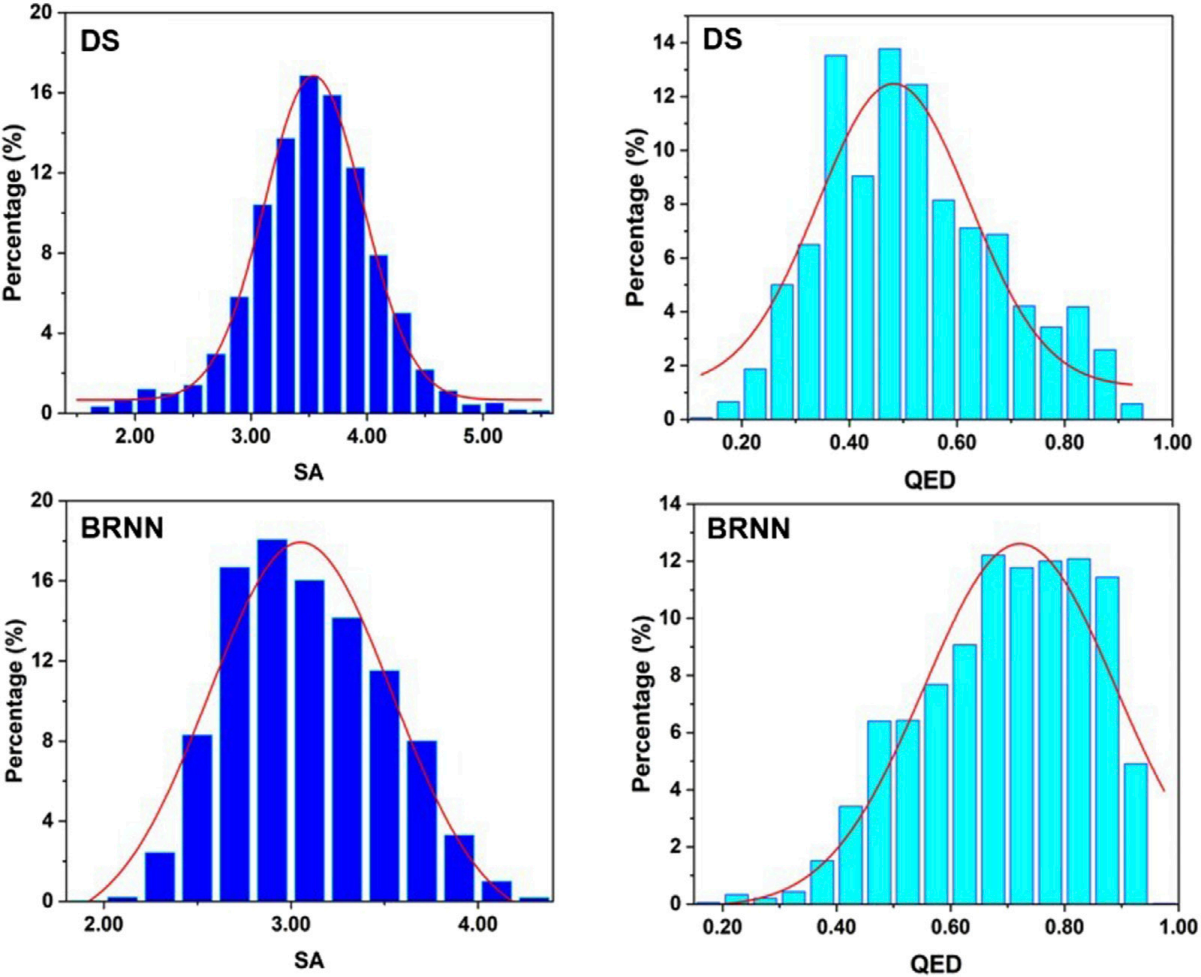
Distribution of QED and SA scores of molecules generated by BRNN and scaffold hopping.

To explore the drug-likeness and synthetic difficulty of the molecules generated by BRNN and scaffold hopping, QED and SA values of these molecules were calculated. It can be seen from Figure 5 that the highest point in the distribution of SA scores of molecules generated by DS is at 3.5, while the highest point in the distribution of SA scores of molecules generated by BRNN is at 2.8, and the SA scores of molecules generated by BRNN are more skewed towards 1 and show a left-skewed distribution, which suggests that molecules generated by BRNN have a better synthesizability. The highest point in the distribution of QED scores of molecules generated by DS is at 0.5 and the overall distribution is skewed towards 0, while the highest point of QED score distribution of molecules generated by BRNN is at 0.65 and the overall distribution is skewed towards 1, indicating that molecules generated by BRNN have higher drug-like properties. Overall, the BRNN-generated molecules have higher synthesizability and drug-like properties relative to the DS-generated molecules.

### 3.5 Redocking validation

**FIGURE 6:**
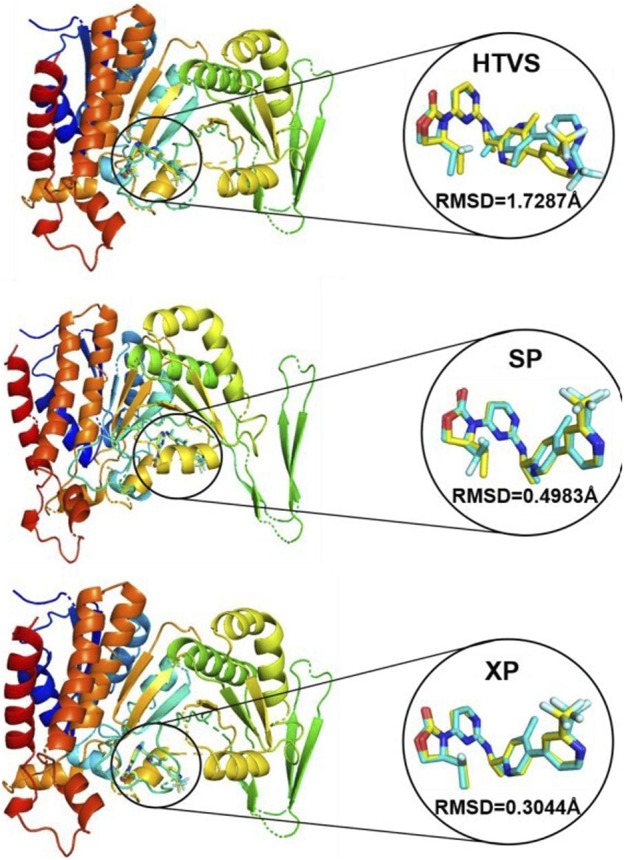
Superposition of conformational proto-ligand and co-crystalline molecular docking conformations.

During the process of HTVS, SP and XP docking modes, the docking scores of the co-crystal IDH305 after sub-stacking were −9.768 kcal/mol, −10.658 kcal/mol and −11.097 kcal/mol, respectively. The RMSD values were 1.7287 Å, 0.4983 Å, 0.3044 Å, which were less than 2 Å (Figure 6), indicating that the docking results were reliable and the Schrödinger 2022 can reproduce the binding mode of the protein and ligand.

### 3.6 Distribution of docking score of molecules

**FIGURE 7:**
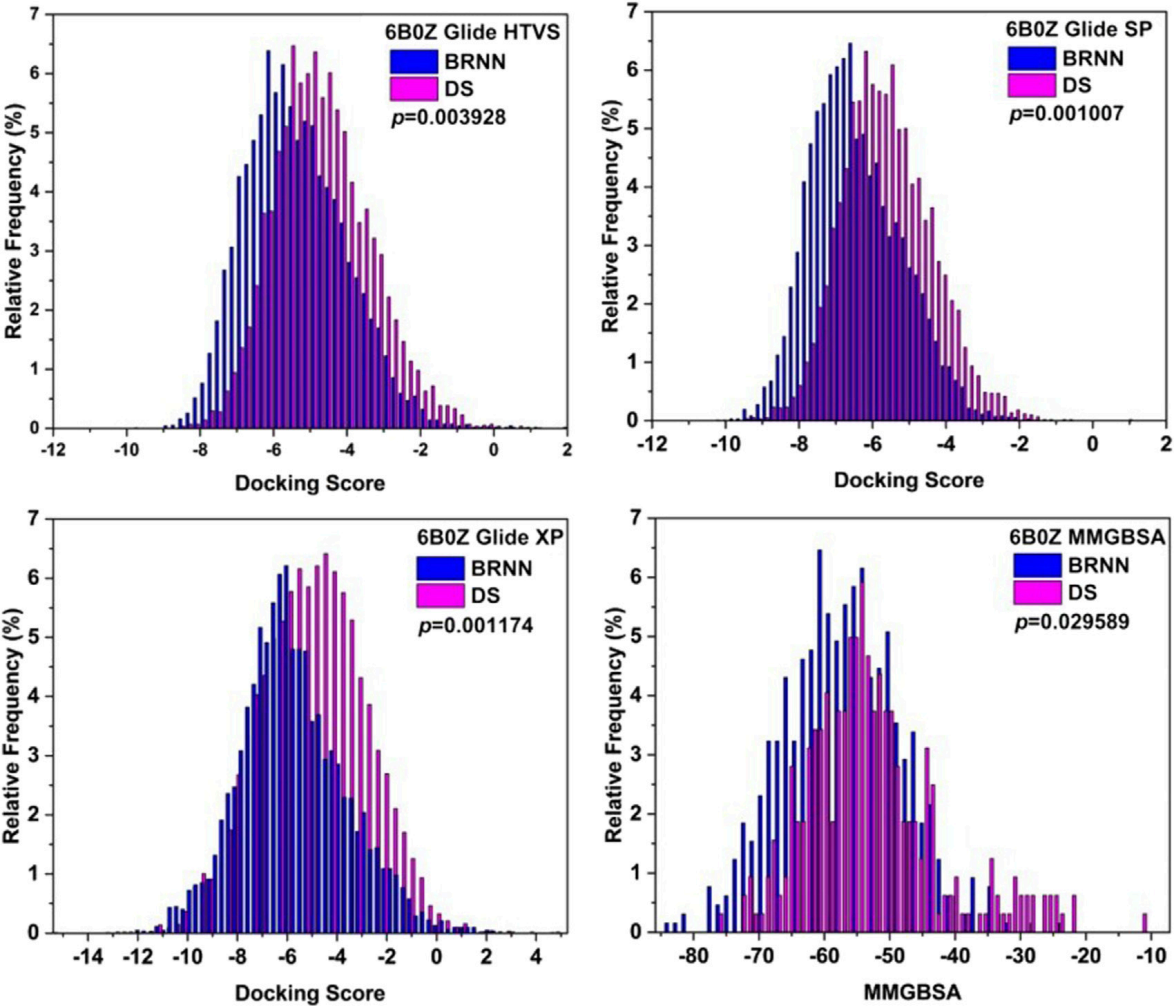
Distributions of Glide docking scores of molecules generated by BRNN and scaffold hopping.

The results of the distribution of docking score of molecules are shown in Figure 7. The molecular scoring distributions of BRNN-generated molecules are more to the left relative to those of DS-generated molecules under the three docking accuracy modes of HTVS, SP and XP, and the P-values of the docking scores of both are less than 0.01, which proves that BRNN-generated molecules have a better docking scores under these three docking accuracies. In order to further compare the differences between the two, the MMGBSA of the XP precision docking results were calculated separately, and the results showed that the molecular binding free energy distribution of BRNN-generated molecules was more to the left relative to that of DS-generated molecules, and the P-value of the binding free energy of the two was less than 0.05, which further proved that the molecules generated by the BRNN model were more promising as potential mIDH1 inhibitors.

### 3.7 Virtual screening of BRNN for mIDH1 inhibitor

**FIGURE 8:**
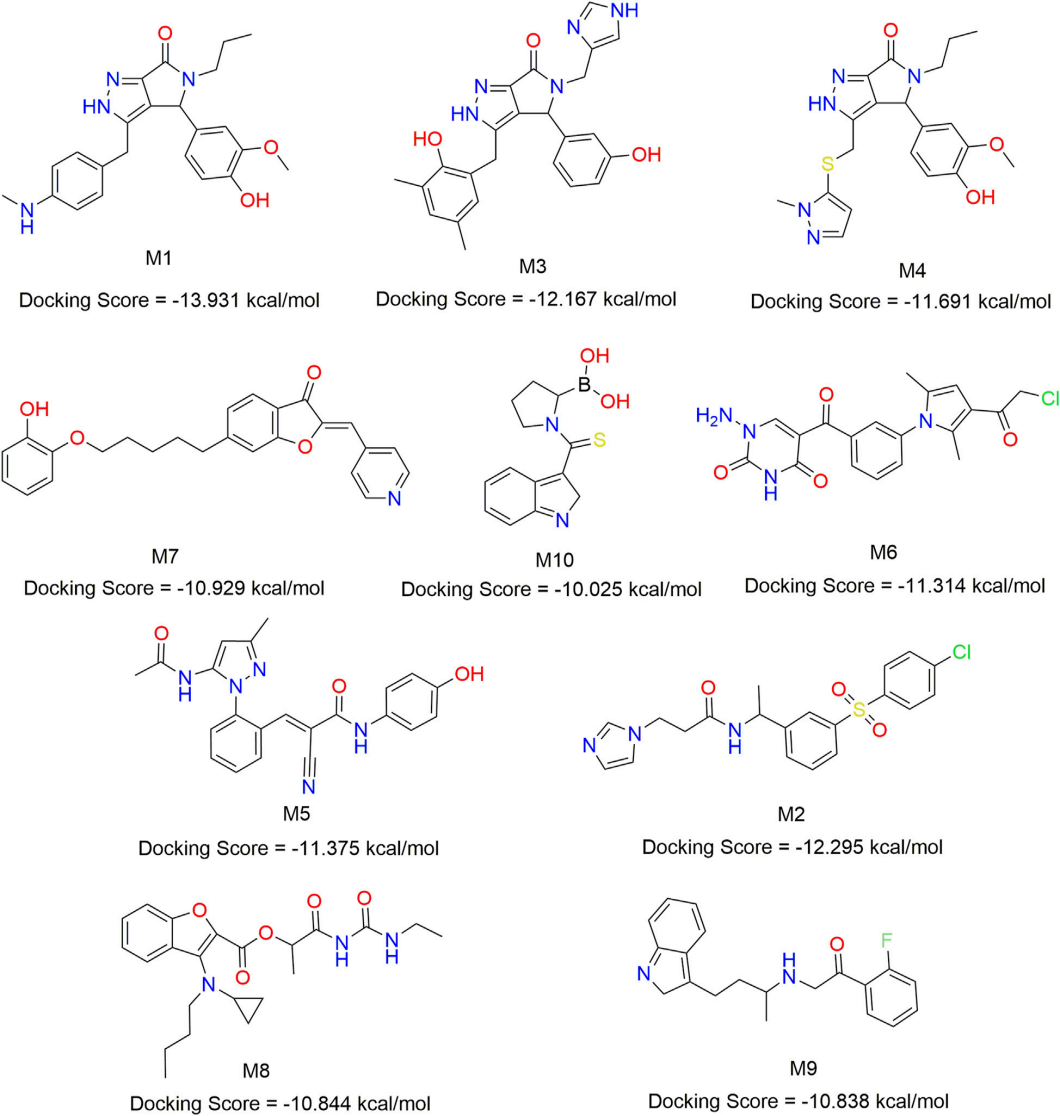
Chemical structures of 10 compounds identified by structural virtual screening and docking scores.

In order to find mIDH1 inhibitors, virtual screening was utilized to uncover the most promising compounds based on docking score. The screening was carried out using three screening processes, HTVS, SP and XP, provided by the VSW module in the Schrödinger software. Firstly, a large-scale virtual screening was carried out using HTVS, retaining the top 30% to obtain 1,167 compounds. Secondly, in order to make the screening results more precise, a molecular docking screening with standard accuracy was performed on the obtained results using the SP calculation module of Glide, and 234 compounds were obtained by retaining the first 20%. In order to obtain more accurate screening results, Glide’s XP calculation module was used to perform more accurate virtual screening of the drugs, retaining the top 10% of the drug molecules. After undergoing a three-step screening procedure that included HTVS, SP, and XP, 21 compounds were discovered with the top docking score. The MM-GBSA analysis was carried out on a group of 21 compounds, with ∆G Bind being chosen as the parameter. Consequently, a total of 10 compounds showing an affinity of below −50 kcal/mol were identified as potential inhibitors (Figure 8). The detailed docking scores are shown in Table 1.

**TABLE 1:** The Docking score, MW and MM-GBSA energy of the top 10 compounds screened.

ID	MW	Docking score (kcal/mol)		
		SP	XP	MMGBSA
IDH305	490.459	−10.658	−11.097	−55.65
M1	406.483	−11.659	−13.931	−70.93
M2	417.909	−11.412	−12.295	−69.02
M3	429.477	−11.163	−12.167	−66.21
M4	413.493	−11. 117	−11.691	−64.19
M5	401.424	−10.939	−11.375	−58.72
M6	400.821	−10.736	−11.314	−57.95
M7	401.461	−10.586	−10.929	−54.49
M8	415.488	−10.533	−10.844	−53.34
M9	324.397	−10.370	−10.838	−52.76
M10	274.091	−10.025	−10.438	−50.51

### 3.8 ADME prediction of small molecules

ADME is a comprehensive study of drug absorption, distribution, metabolism and excretion, which is an important method to study the *in vivo* processes of drugs and an important criterion to be considered in drug screening (Zhang et al., 2024). The information in Table 2 shows that CNS ranges from −2 to 0, QPlogPo/w values range from 2.795 to 5.026, QPlogPC16 values range from 11.474 to 15.411, and QPlogPoct values range from 16.543 to 25.911. In addition, QplogPw ranges from 8.954 to 16.834. QPlogS values ranged from −3.33 to −7.55. CIQPlogS values ranged from −3.77 to −8.73 and QplogHERG values ranged from −4.014 to −7.665. The human oral absorption values ranged from 1 to 3, with a percentage range of 73.275%–100%. The ADME results indicated that the selected compounds exhibit suitable oral absorption capacity, along with appropriate solubility and absorption characteristics in line with drug-like principles and low central nervous system activity. Human intestinal absorption is critical for determining drug bioavailability, and *in vitro* models such as Caco-2 and MDCK cell lines are widely used to assess intestinal drug absorption and blood-brain barrier permeability. The QPlogKp of these molecules ranges from −1.413 to −4.812, the QPPCaco ranges from 52.306 to 197.299, the QPlogBB ranges from −0.157 to −2.169, the QPPMDCK ranges from 32.653 to 368.919, the #Metab ranges from two to eight, the QPlogKhsa ranged from 0.146 to 0.783, indicating that these molecules have high intestinal, skin and blood-brain barrier permeability with high solubility and bioavailability.

**TABLE 2:** The ADME prediction of the top 10 compounds screened.

ID	IDH305	M1	M2	M3	M4	M5	M6	M7	M8	M9	M10
CNS	−1	−2	−1	−2	−2	−2	−2	−2	0	1	−2
QPlogPo/w	4.881	4.037	2.787	2.795	4.181	2.611	2.511	5.026	3.872	3.643	2.954
QPlogPC16	13.287	13.569	13.022	15.036	13.224	14.904	13.133	15.411	13.7	11.474	13.036
QPlogPoct	23.705	21.067	20.762	25.911	19.338	24.21	21.264	19.855	20.098	16.543	22.145
QPlogPw	12.267	10.834	13.67	16.834	9.196	16.227	13.169	10.331	10.795	8.954	12.845
QPlogS	−7.549	−6.269	−3.516	−5.635	−6.201	−6.472	−5.634	−6.773	−5.049	−3.926	−4.135
CIQPlogS	−7.221	−6.346	−4.677	−6.181	−6.497	−6.045	−5.551	−6.436	−3.918	−3.601	−6.82
QPlogHERG	−6.383	−5.782	−4.087	−6.189	−5.472	−6.777	−5.606	−7.665	−5.562	−6.947	−4.014
QPPCaco	87.533	197.299	117.795	80.89	85.456	102.813	58.511	52.306	142.573	86.717	117.924
QPlogBB	−0.638	−1.749	−0.461	−2.116	−1.673	−2.169	−1.931	−1.605	−0.842	−0.157	−1.457
QPPMDCK	286.901	85.598	205.874	32.653	103.276	42.315	57.105	245.673	111.114	368.919	49.075
QPlogKp	−2.3	−3.46	−1.303	−4.001	−3.598	−3.495	−4.812	−1.413	−4.151	−3.14	−4.01
#Metab	5	6	2	8	4	2	3	6	3	4	8
QPlogKhsa	0.641	0.751	−0.367	0.348	0.783	0.146	0.273	0.753	0.535	0.369	0.461
Human Oral Absorption	1	1	3	2	1	1	2	1	3	3	2
Percent Human Oral Absorption	100	91.661	100	77.457	92.024	78.245	73.275	92.073	88.172	95.879	81.319

### 3.9 RMSD/RMSF analysis

**FIGURE 9:**
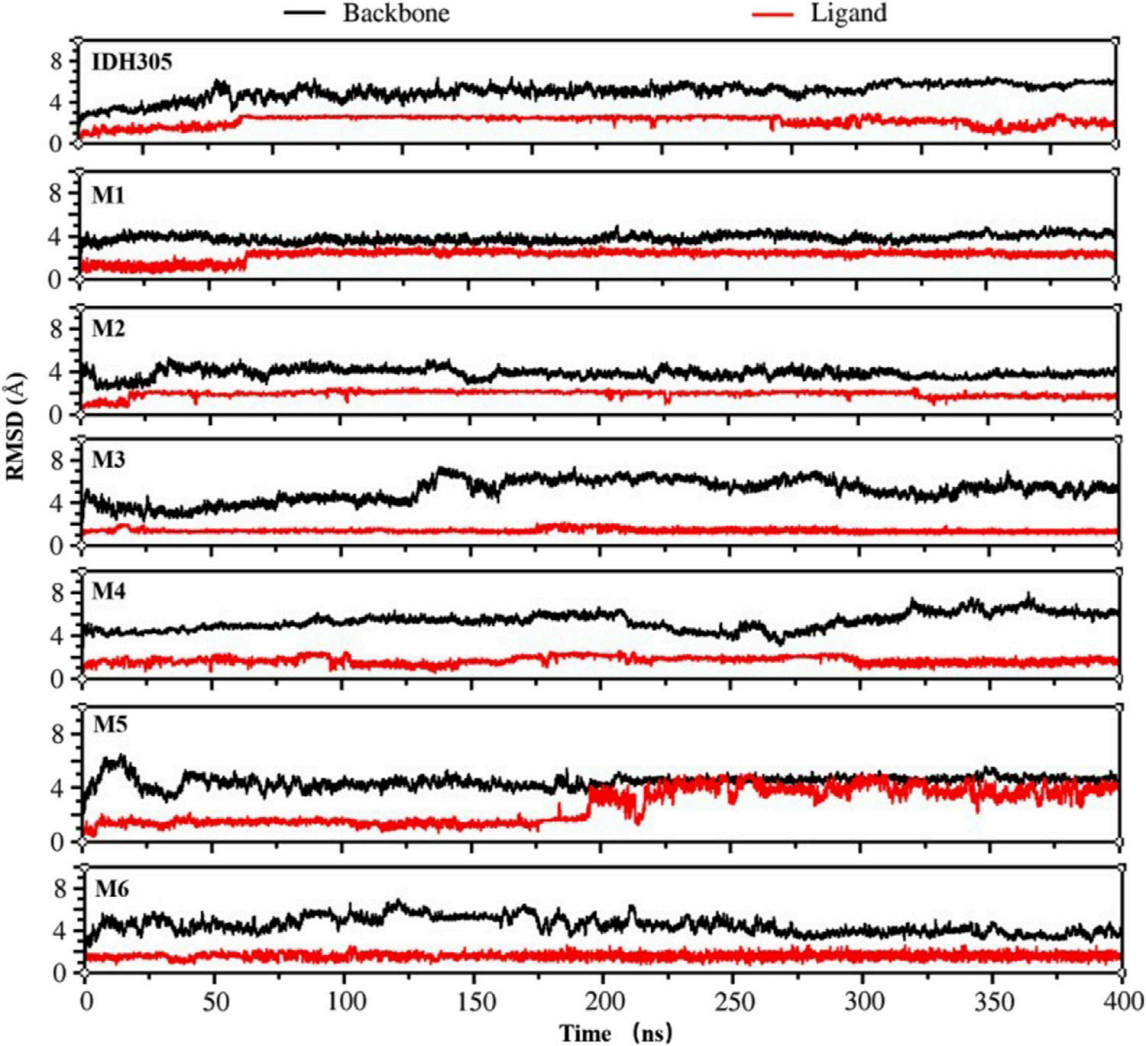
Fluctuation of RMSD values for IDH305 and six molecules during 400 ns MD simulations.

**FIGURE 10:**
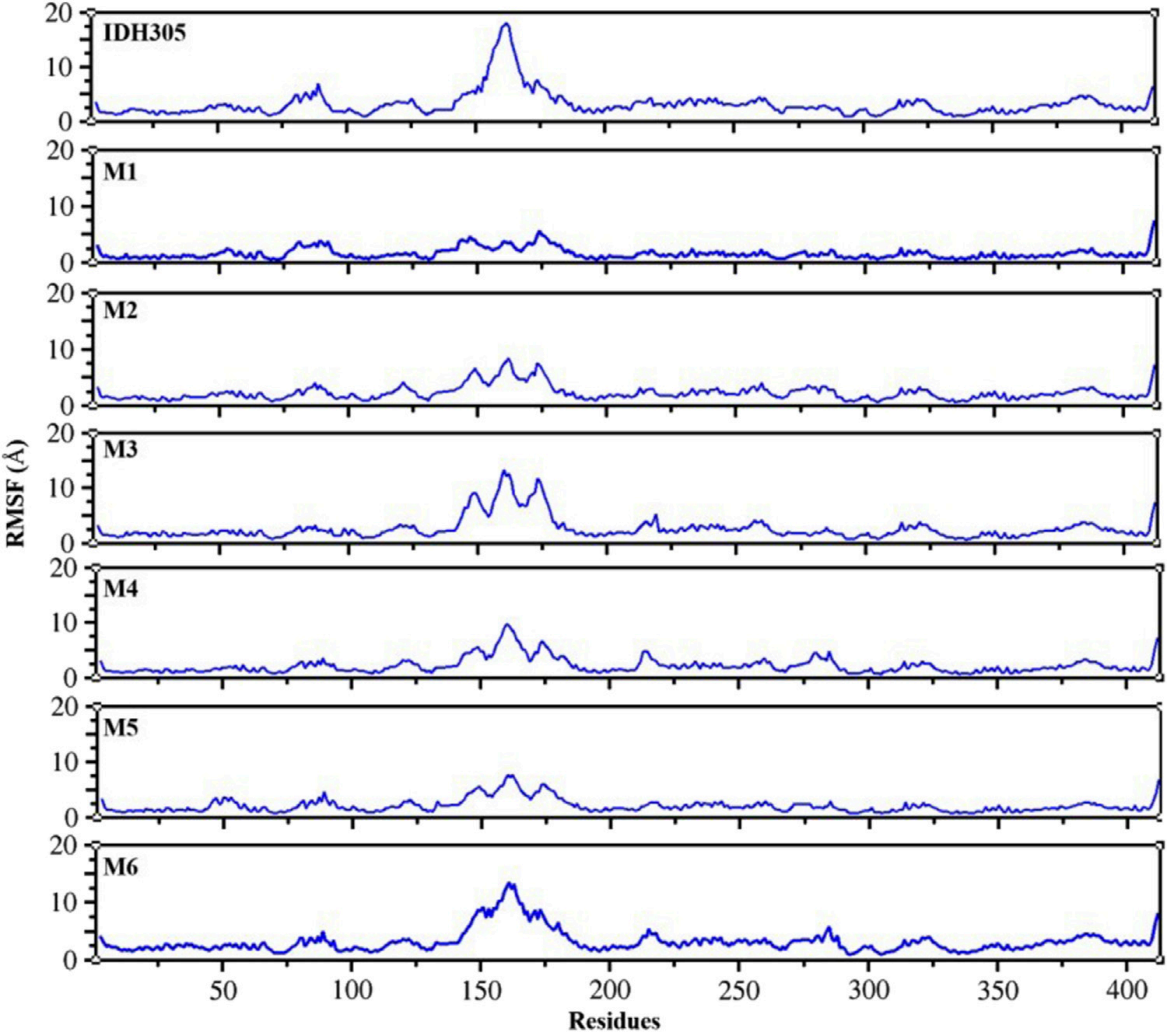
Fluctuation RMSF values for IDH305 and six molecules during 400 ns MD simulations.

To gain the detailed structural basis, molecular dynamics simulations were performed. RMSD is commonly utilized for assessing the stability and dynamics of the overall molecular structure (Gogoi et al., 2021). The simulation ran for 400 ns, during which the stability of mIDH1 backbone and ligands was assessed through RMSD calculations. As depicted in Figure 9, compounds IDH305, M1 and M2 reached stability after 200 ns, and the RMSD fluctuations of both Backbone and Ligand remained within 2 Å, indicating that the three compounds had high overall system stability during the simulation. Among them, compounds M1 and M2 showed lower RMSD amplitudes compared to the active compound IDH305, indicating that compounds M1 and M2 possessed higher kinetic stability properties than the positive drug IDH305. In contrast, compounds M3, M4 and M6 showed more pronounced Ligand stability throughout the kinetic simulation, indicating that the three compounds were more stable at their binding sites. M5 had better Backbone stability during the simulation, but Ligand stability was lower compared to the other compounds, probably due to the conformational change of compound M5 during the simulation. RMSF analysis is a widely used technique for evaluating local flexibility and residue fluctuation (Alamri et al., 2021). Simulation results indicated that the RMSF values of these five systems displayed similar trends, highlighting the dynamic impact of inhibitors on protein interiors. As can be seen in Figure 10, the RMSF values of most residues in the seven systems fluctuated within the range of 2 Å, indicating that these regions were relatively stable during the simulation, but there were some regions that showed higher fluctuations, for example, significant differences in RMSF values (greater than 2 Å) were observed in the regions of residues 75–100, 125–200 and 275–300. These regions may be flexible regions of the protein and may correspond to functionally relevant active sites. These regions are mainly located near the binding site, suggesting that there are large conformational changes as well as higher flexibility in this region. Larger conformational changes may have an important effect on ligand binding and dissociation, and higher flexibility is usually associated with compactness and reduction of intramolecular hydrogen bonding, which may affect the distances between key residues. M1, M2 and M5 have the lowest RMSF values among the complexes, indicating that these three complex systems have better binding stability during the simulations.

### 3.10 Radius of gyration (Rg), hydrogen bond and Solvent Accessible Surface Area (SASA) analysis

**FIGURE 11:**
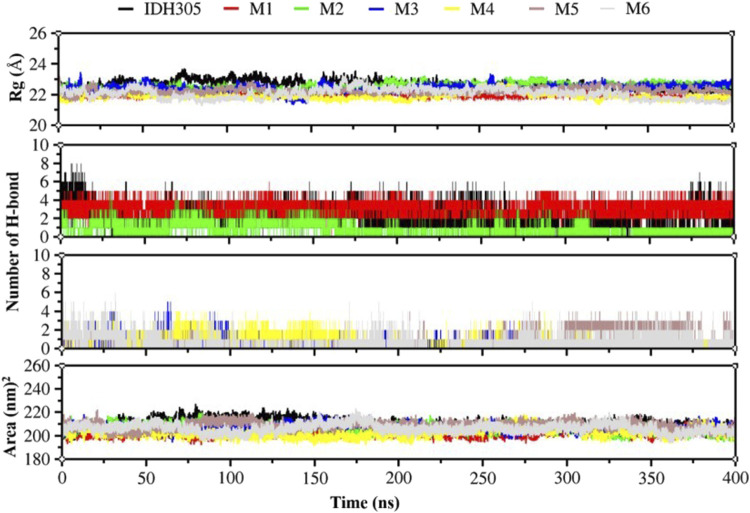
Rg values, hydrogen bonding interactions and Area values for compounds IDH305, M1, M2, M3, M4, M5 and M6 for 400 ns MD simulation time.

The radius of gyration (Rg) is commonly utilized to evaluate the overall compactness and structural evolution, serving as a key parameter in assessing the conformational properties (Patel C. N. et al., 2020). As depicted in Figure 11, the Rg values for these seven complexes remained consistent during 400 ns dynamic simulations, suggesting a level of compactness similar to that of the IDH305.

H-bond analysis was performed to examine the hydrogen bonding in complexes over the 400 ns simulation period, as depicted in Figure 11. The number of hydrogen bonds in M1 is stable at 4 during the simulation, and the number of hydrogen bonds in M2, M3,M4, M5 and M6 ranges from 0-4Å during the simulation. The number of hydrogen bonds in IDH305 reaches up to 8 during the simulation at 400 ns?

The SASA was theoretically utilized as a parameter for characterizing the ratio of protein-solvent interaction, which can predict the extent of conformational change during the binding process and assess protein accessibility (Vivek-Ananth et al., 2021). The gmx sasa program was employed to calculate SASA, and the outcomes are depicted in Figure 11. The SASA of IDH305, M1, M2, M3, M4 and M6 fluctuated within the range of 200–215, 200–190, 195–215, 195–225, 196–223 and 195–225 nm^2^, and the overall fluctuation was relatively smooth, indicating that the molecular structure was relatively stable. While the SASA of M5 had a decreasing trend during the molecular dynamics simulation. Among them, the SASA of M5 rapidly decreased from 220 to 185 nm^2^ at 100 ns, which indicated that the interaction between the surface of the mIDH1 protein and the solvent was reduced at this time, and the small molecule might have undergone a conformational change or conformational rearrangement.

### 3.11 Dynamic cross-correlation maps and free energy landscape

**FIGURE 12:**
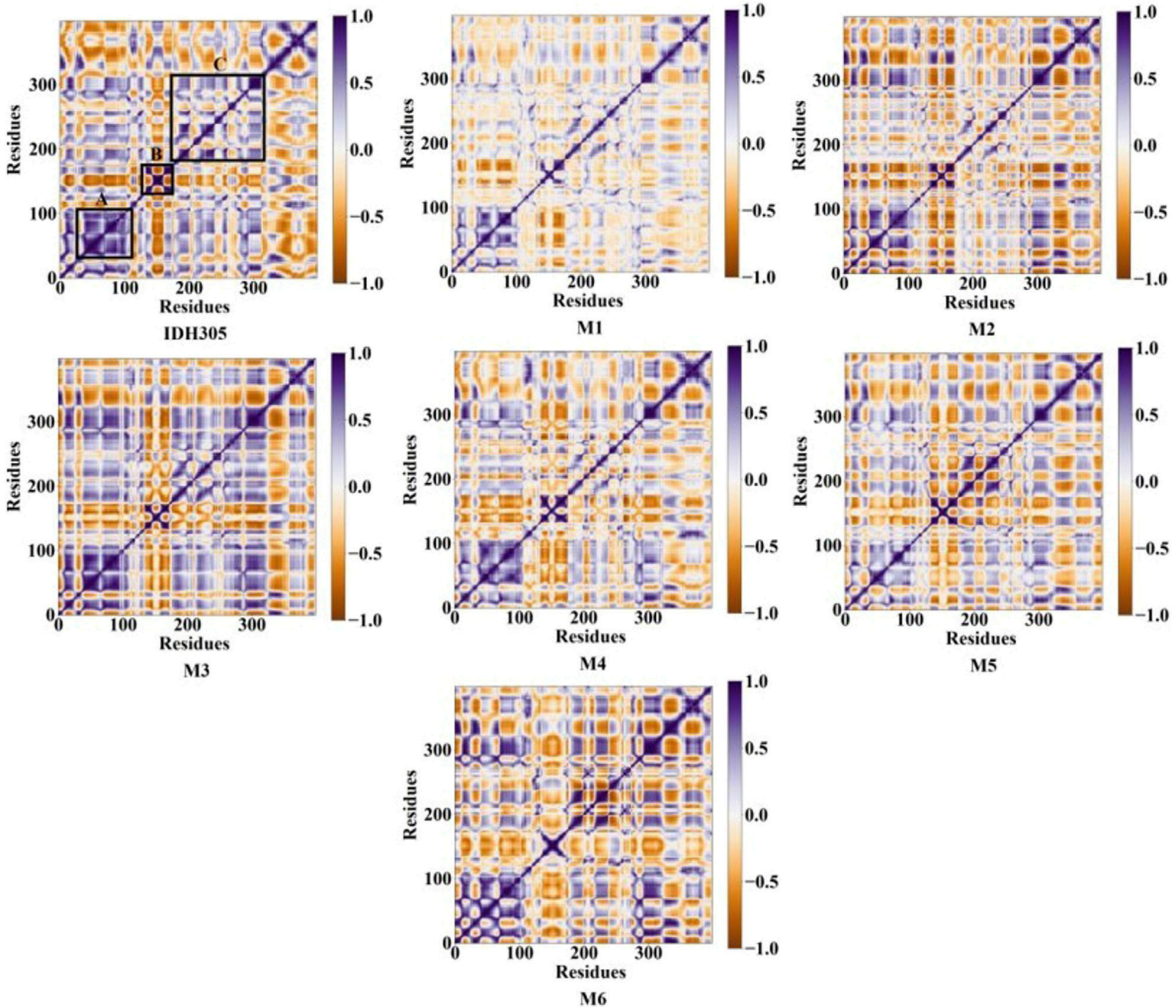
DCCM of the six molecules and IDH305.

The gmx covar command in the GROMACS 2022 software was utilized to extract the C-alpha coordinates of MD trajectories, and DCCMs graphs were generated for investigating the dynamic interactions between inhibitors and mIDH1. Figure 12 illustrates the correlated motions among residues in seven systems, with blue-purple regions denoting positive correlation and brown regions indicating negative correlation. The correlation values range from −1 to 1, where values falling between −0.25 and 0.25 signify low correlation. The diagonal primarily depicts positively correlated motions within individual residues, while the off-diagonal region mainly reveals reverse correlations or cooperative actions between residues. Alterations in these patterns are especially conspicuous within the regions demarcated by black boxes. The diagonal elements of part A, part B and part C region in IDH305, M1, M2, M3, M4, M5 and M6 show a strong positive correlation among residues. Compared to IDH305, the binding of M1, M2, M4, M5 and M6 notably weakened the positive correlation motion in part A while weakening it significantly in parts B for M1, M4 and M6. Additionally, M3 and M6 notably increased positive correlation motion in part C. These observed significant changes corresponded to previous RMSF findings with in part B. Therefore, distinct substitutions at the same position can induce variations in mDIH1 internal dynamics.

**FIGURE 13:**
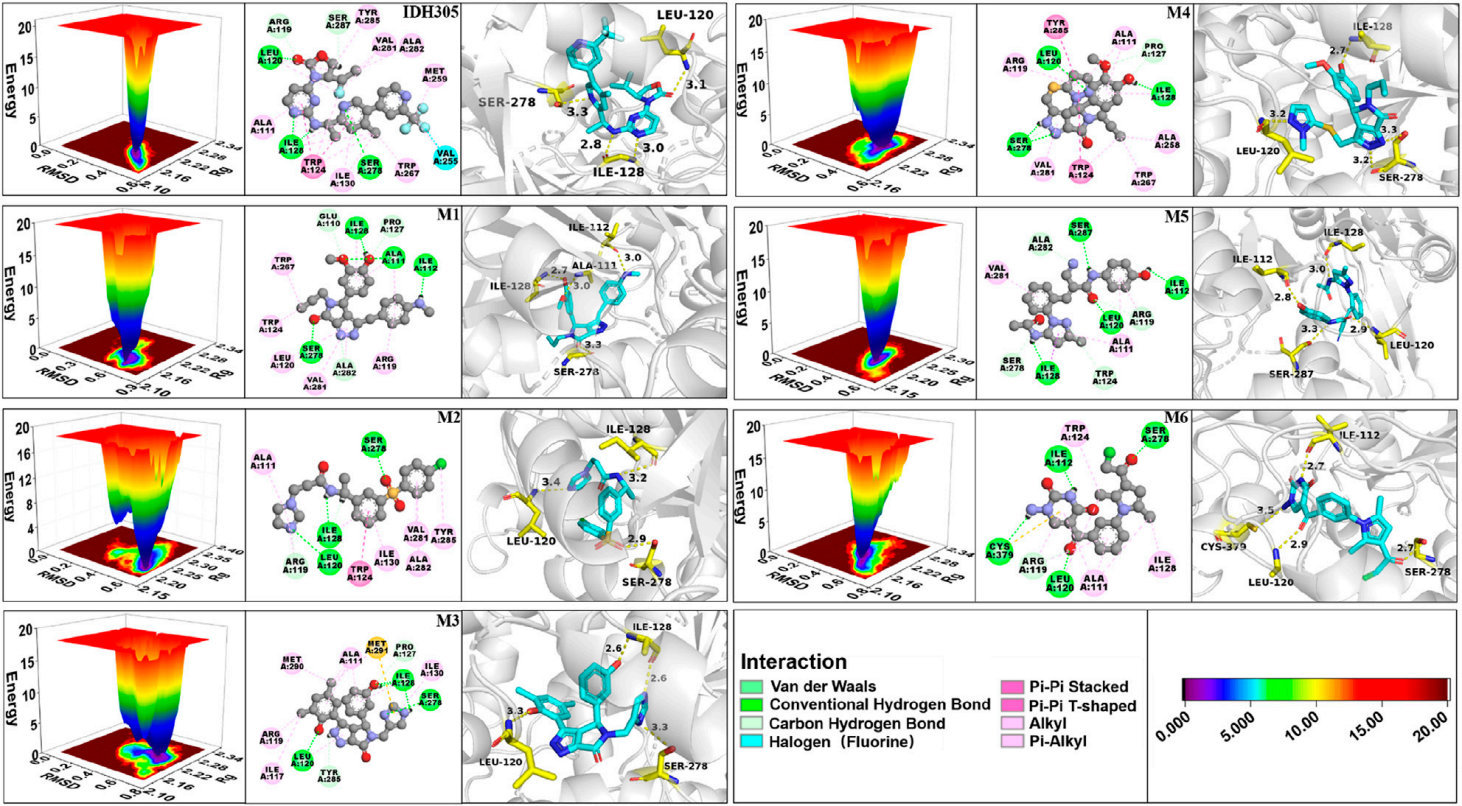
3D free energy landscape, 2D interaction and 3D interaction diagrams for IDH305, M1, M2, M3, M4, M5 and M6.

FEL is a tool for visualizing the energy-structure relationship of proteins. Figure 13 illustrates the 3D free energy landscape, 2D binding mode and 3D binding mode maps for the seven mIDH1 complex systems. The lowest point in the 3D free energy landscape represents the lowest energy state or conformation of the mIDH1 system. The conformational transitions within each complex are delineated by a subspace, indicating that these small molecule inhibitors bind to the protein through different binding modes, resulting in minimal binding effects. Representative conformations during the simulations were chosen based on these principal components. The 3D and 2D binding patterns between IDH305 and mIDH1 indicate that a hydrogen bond is formed between the carbonyl group in the molecule and LEU120, another hydrogen bond is formed between the nitrogen atom in the pyrimidine ring and ILE128, and a third hydrogen bond is formed between the nitrogen atom in the pyridine ring and SER278. Additionally, TRP124 interacts with the pyridine and pyrimidine rings through π-π stacking. The binding mode of M1 and mIDH1 leads to a significant shift in the position of the carbon-oxygen bond, causing a notable change in the docking orientation of the small molecule. In particular, SER278 forms a hydrogen bond with the carbonyl, anisole interacts with ALA111 through a hydrogen bond, ILE128 engages in a hydrogen bond interaction with the phenolic hydroxyl, and a set of hydrogen bonds is formed between ILE112 and the amino group. In the interaction between M2 and mIDH1, a series of hydrogen bonds are formed with LEU120 by the nitrogen atom in the imidazole ring, with ILE128 by the amide group, and with SER278 by the oxygen atom of the sulfonyl group. Additionally, there is a π-π stacked interaction between the benzene ring and TRP124. In the binding mode of M3 and mIDH1, ILE128 forms hydrogen bonds with the two nitrogen atoms of the thiazole ring, while SER278 forms a hydrogen bond with one nitrogen atom. Furthermore, ILE128 also interacts with the phenolic hydroxyl group through a set of hydrogen bonding interactions. Additionally, a hydrogen bond interaction is formed between LEU and another phenolic hydroxyl group of M3. In the binding mode of M4 and mIDH1, SER278 forms a hydrogen bond with the nitrogen atom of the pyrrole ring, LEU120 forms a hydrogen bond with the nitrogen atom in the pyrazole ring, and ILE128 forms a set of hydrogen bonds with anisole. Additionally, TRP124 and TYR285 each engage in π-π stacked interactions with the benzene ring. In the binding mode of M5 and mIDH1, LEU120 forms hydrogen bonds with the carbonyl group, ILE112 forms hydrogen bonds with the phenolic hydroxyl group, SER287 forms hydrogen bonds with the nitrogen atoms of the amide, and ILE128 forms hydrogen bonds with another set of amides of M5. In the binding mode of M6 and mIDH1, the carbonyl group forms hydrogen bonds with SER278 and LEU120, respectively, while CYS379 forms a hydrogen bond with the amino. In addition, the nitrogen atom in the pyrimidine ring forms a set of hydrogen bonds with ILE112. By comparing the interaction patterns of IDH305, we found that M2, M3, M4, M6 have a set of hydrogen-bonding interactions with the key amino acids ILE128, SER278 and ILU120 with a distance of less than 3.5 Å. Six small molecules have a similar binding interaction pattern to IDH305, and the similar interaction pattern may be the reason for the higher affinity. Furthermore, the binding modes of M1, M2, M3, M4, M5 and M6 with mIDH1 exhibited a higher level of interaction with the active site in comparison to IDH305. This finding is in line with the results obtained from their binding affinity analyses.

### 3.12 Analysis of binding free energy

The calculation of the free energy of intermolecular binding and the resulting inhibition constant (Ki) are commonly used to assess the strength of the interaction between a molecule (e.g., a drug molecule and a target protein). The ΔG_bind_ of IDH305, M1, M2, M3, M4, M5 and M6 were −19.817 kcal/mol, −30.718 kcal/mol, −28.241 kcal/mol, −22.717 kcal/mol, −22.399 kcal/mol, −22.369 kcal/mol and −21.860 kcal/mol, respectively (as shown in Table 3). In these complexes, M1 possessed the optimal binding affinity, and all complexes showed better binding affinity than the positive control IDH305 (−19.817 kcal/mol), consistent with the molecular docking results. The van der Waals energy (∆Evdw) values were −43.768 kcal/mol, −46 kcal/mol, −42.583 kcal/mol, −44.93 kcal/mol, −37.799 kcal/mol, −44.847 kcal/mol and −46.047 kcal/mol, respectively, indicating the positive role of van der Waals interactions in ligand binding. ΔE_SA_ represents the solvable surface area, where M1 has the smallest solvable surface area and is the most favorable for stable binding. The Ki value is used to quantify the affinity or binding strength of an inhibitor, and in this study, the Ki value for M1 was the smallest of the six compounds at 3.045E-14, which suggests that M1 binds most strongly to the receptor and is the most potent.

**TABLE 3:** MM/PBSA binding Free energy (kcal/mol) analysis of the hit compound’s complexes.

Energy terms (Kcal/mol)	IDH305	M1	M2	M3	M4	M5	M6
ΔG/ΔG_bind_	−19.817 ± 3.874	−30.718 ± 1.672	−28.241 ± 1.373	−22.717 ± 2.962	−22.399 ± 2.491	−22.369 ± 2.96	−21.86 ± 1.448
ΔH/ΔE_Binding_	−21.204 ± 3.874	−32.634 ± 1.672	−30.997 ± 1.373	−24.732 ± 2.962	−25.469 ± 2.491	−24.843 ± 2.96	−25.465 ± 1.448
Ki (nM)	2.98E-06	3.045E-14	1.99E-12	2.23E-08	3.81E-11	4.01E-08	9.48E-08
−TΔS	1.387	1.9161	2.755	2.015	3.070	2.474	3.605
ΔE_VDW_	−43.768 ± 2.855	−46 ± 1.013	−42.583 ± 2.178	−44.93 ± 1.54	−37.799 ± 2.253	−44.847 ± 3.383	−46.047 ± 1.624
ΔE_COU_	−18.055 ± 2.552	−10.494 ± 1.214	−11.782 ± 2.206	−6.52 ± 0.87	−5.923 ± 1.461	−3.264 ± 2.379	−7.005 ± 1.084
ΔE_MM_	−61.823 ± 1.626	−56.494 ± 1.794	−54.365 ± 2.619	−51.45 ± 2.346	−43.722 ± 2.805	−48.111 ± 3.625	−53.052 ± 2.569
ΔE_PB_	47.133 ± 2.976	31.171 ± 1.493	29.766 ± 2.084	33.681 ± 1.623	24.357 ± 2.328	29.808 ± 2.179	34.64 ± 2.002
ΔE_SA_	−6.514 ± 0.198	−7.311 ± 0.141	−6.398 ± 0.167	−6.963 ± 0.144	−6.104 ± 0.426	−6.54 ± 0.071	−7.052 ± 0.192
ΔE_PBcom_	−3135.426 ± 43.007	−3316.136 ± 102.502	−3310.787 ± 80.483	−3253.908 ± 117.952	−3274.957 ± 88.882	−3426.912 ± 31.894	−3,348.73 ± 82.995
ΔE_PBpro_	−3145.018 ± 43.997	−3322.892 ± 103.359	−3307.11 ± 80.62	−3251.864 ± 117.302	−3268.931 ± 89.265	−3433.222 ± 31.742	−3350.214 ± 80.941
ΔE_PBlig_	−37.541 ± 1.023	−24.415 ± 0.605	−33.444 ± 0.94	−35.725 ± 1.483	−30.383 ± 1.392	−23.498 ± 1.603	−33.156 ± 1.514
ΔE_SAcom_	144.111 ± 1.624	143.705 ± 1.253	147.166 ± 1.21	146.995 ± 1.322	145.804 ± 2.951	148.143 ± 1.47	145.449 ± 1.09
ΔE_SApro_	146.028 ± 1.636	145.861 ± 1.33	148.884 ± 1.142	149.159 ± 1.345	147.234 ± 2.684	150.291 ± 1.395	147.689 ± 1.031
ΔE_SAlig_	4.597 ± 0.054	5.155 ± 0.099	4.68 ± 0.085	4.798 ± 0.075	4.674 ± 0.091	4.391 ± 0.064	4.812 ± 0.1

**FIGURE 14:**
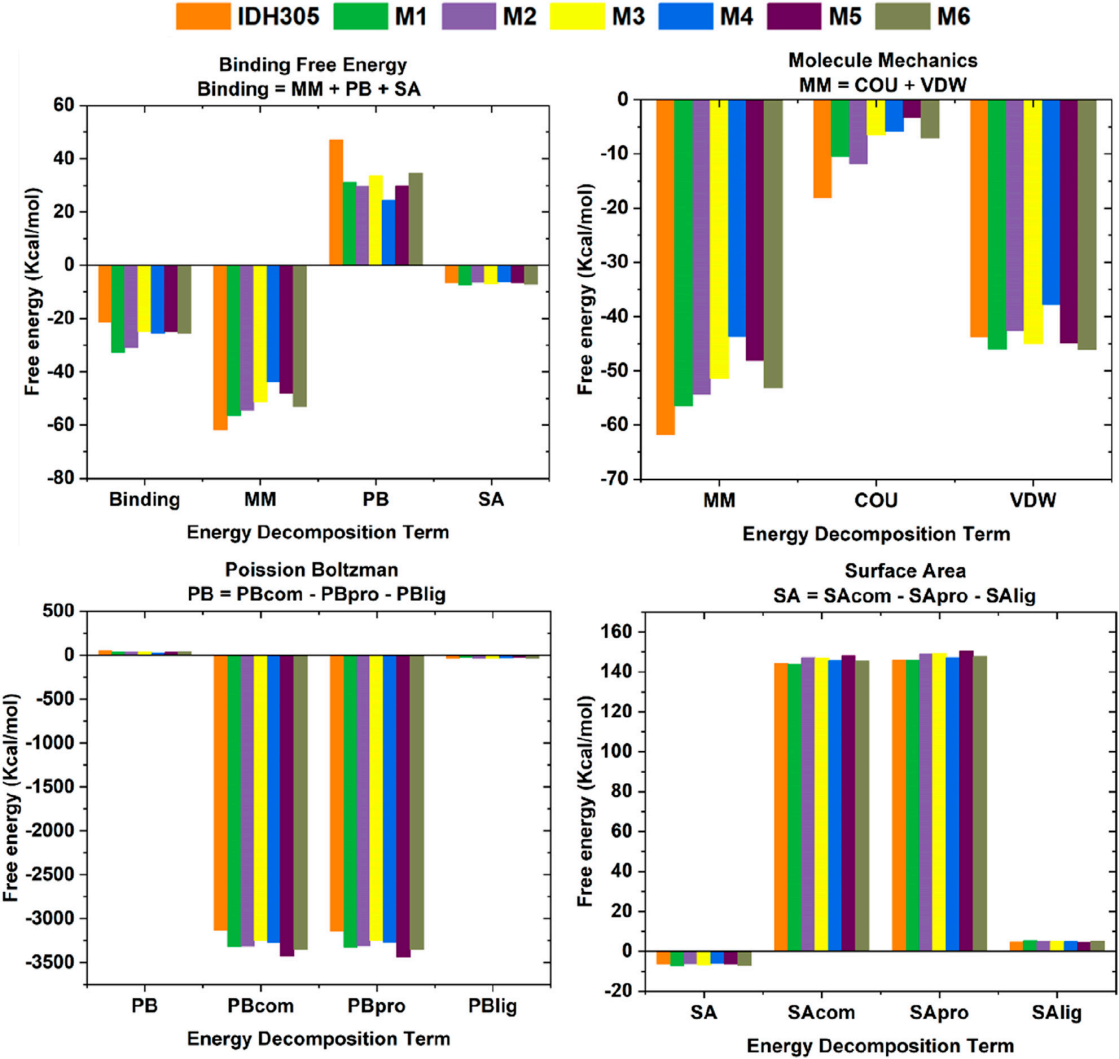
Comparison of ligand energies for IDH305, M1, M2, M3, M4 and M5.

In this research, histograms were used to visualize the binding free energy (Binding), molecular mechanics (MM), Poisson Boltzman (PB) and Solvent Accessible Surface Area values of the seven compounds (Figure 14). It was visualized that M1 had the best binding among the seven small molecules and all the six candidate small molecules had better binding free energies than the positive drug IDH305. The six small molecules exhibited higher energies in MM than the positive drug, which may imply that they have stronger electrostatic and hydrophobic interactions with IDH1 protein. The seven small molecules showed a small difference in PB values varied less and were all lower, the lower ΔE_PB_ may indicate more favorable electrostatic interactions between the molecules and the IDH1 protein, thus enhancing the binding stability and affinity. The SA values of the seven small molecules varied less and were all negative, suggesting that all of them had a smaller solvent-accessible surface area with the target solvent molecules.

### 3.13 Energy decomposition diagrams for per residue

**FIGURE 15:**
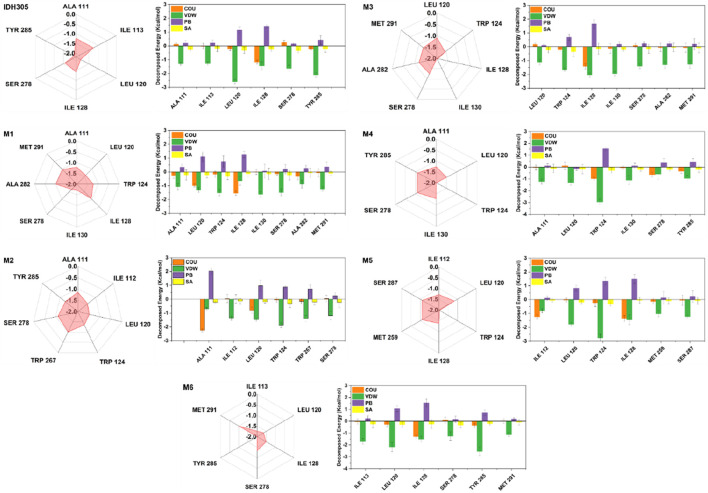
Key residues energy decomposition diagram of the IDH305, M1, M2, M3, M4, M5 and M6.

In this study, we further investigated the interaction energies of IDH305, M1, M2, M3, M4, M5 and M6 in order to gain a more comprehensive understanding of their interactions. Specifically, we examined the residue interaction energies that were greater than 1.0 kcal/mol in terms of Coulombic Interactions, polar solvation free energy (PB), nonpolar solvation free energy (SA) and van der Waals interactions (VDW), as shown in Figure 15. The IDH305 system shows the importance of certain residues, including ALA111, ILE113, LEU120, ILE128, SER278 and TYR285. Van der Waals interactions play a critical role in the binding of ligands, while the polar effect negatively impacts this binding process. The carbonyl group of IDH305 is involved in a hydrogen bond with LEU120, the nitrogen atom in the pyrimidine ring forms a hydrogen bond with ILE128, and the nitrogen atom in the pyridine ring forms a hydrogen bond with SER278. Furthermore, π-π stacking interactions are observed between TRP124 and the pyridine and pyrimidine rings.

The interaction energy of M1 with mIDH1 exceeds 1.0 kcal/mol, which is mainly attributed to the van der Waals interactions of M1 with seven specific residues: ALA111, LEU120, TRP124, ILE130, SER278, ALA282 and MET291. In the M2 system, ALA111 interacts with M5 mainly from electrostatic forces, and forms p-alkyl interactions with the benzene ring of M2. Interactions and forms p-alkyl interactions with the benzene ring of M2. The interaction energies of ILE112, LEU120, TPR124, TRP267 and SER278 with M2 are mainly from van der Waals interactions. In the M3, M4 and M6 systems, the interaction forces between the amino acids and the molecules are mainly from van der Waals interactions. In the M5 system, the interaction force between ILE112 and M5 is mainly due to Coulombic energy. The interaction energies of ILE112, LEU120, TPR124, ILE128, MET259 and SER278 with M5 are mainly due to van der Waals interactions.

## 4 Discussion

IDH1 mutations occur in about 20%–30% of gliomas and are a promising target for the treatment of cancer. In recent years, the development of inhibitors targeting IDH1 mutations has become a research hotspot in cancer therapy. The mIDH1 inhibitors inhibit tumor growth by blocking mutant IDH1 enzyme activity, reducing 2-HG production and restoring normal epigenetic regulation. However, although existing IDH1 inhibitors have shown promising therapeutic effects, they have certain limitations, such as the development of drug resistance, adverse reactions, and effects on wild-type IDH1. Therefore, the development of a new generation of mIDH1 inhibitors that are more effective, more selective, and with lower side effects remains one of the important directions of current medicinal chemistry research.

In this study, a series of potential inhibitors against mIDH1 were successfully designed by combining BRNN model, scaffold hopping, molecular docking and molecular dynamics simulations. The BRNN model and scaffold hopping were used to generate 3,890 and 3,680 small molecules, respectively. PCA of the generated small molecules revealed that the molecular distributions generated by the BRNN model and the molecular distributions generated by DS showed some overlap as well as obvious clustering patterns. This distribution model indicates that the two groups of compounds show some similarity in chemical and physical properties. And overall, the molecules generated by BRNN cover a larger area, indicating that the molecular diversity of BRNN-generated molecules is higher than that of DS-generated molecules. The reason for the similarity between molecules generated by the BRNN model and those generated by DS may be due to the fact that they optimize the same batch of molecules. BRNN, as a kind of AI model, can sufficiently learn the patterns and regularities in the large number of molecule data in the training stage, which makes it to innovate based on these patterns when generating new molecules, rather than simply copying known structures. The DS skeleton leaping approach usually starts from a known active molecule and searches for new compounds by changing the parts of the molecule that need to be modified. This approach, although it can produce a range of derivatives related to the original molecule, may limit the range and diversity of generated molecules due to its reliance on predetermined rules of skeleton leaps. QED and SA results indicate that BRNN-generated molecules have superior synthesizability and drug-like properties relative to DS-generated molecules. This may be attributed to the ability of the BRNN model to capture the complex patterns and interactions of the molecules more efficiently when learning from a large number of known active compounds, resulting in the generation of molecules with higher scores in terms of synthetic acceptability and drug-like properties.

In order to ensure the accuracy of the virtual screening, nine crystal structures of mIDH1 (PDB ID: 4UMX, 5L57, 5L58, 5LGE, 5SUN, 5SVF, 5TQH, 6ADG, 6B0Z) were obtained from the PDB database, and the docking and enrichment ability of each crystal structure were evaluated by using multi-target cross-docking. The complex crystal (PDBID: 6B0Z) structure with the best enrichment ability was finally selected as the target. The study was carried out by superimposing the docked conformation of the complex crystalline molecule (IDH305) with the original complex ligand conformation and calculating the RMSD value to show the reliability of the results obtained based on molecular docking screening. The results showed that the ligand docked conformation and the co-crystalline conformation were almost completely overlapped with the RMSD < 2Å, indicating that the virtual screening model constructed in this study has a high degree of reliability, and that Schrödinger software is suitable for this system.

The 3,890 small molecules generated by BRNN were screened using three screening processes provided by the VSW module in the Schrödinger software, resulting in 21 compounds. Subsequently, MM-GBSA analysis was performed to identify 10 candidate molecules with binding affinities lower than −50 kcal/mol. ADME prediction results identified 10 candidate molecules all exhibited positive ADME characteristics to be potential drug candidates, including good lipophilicity, water-solubility balance, and high intestinal and oral absorption rates, further illustrating the applicability of the BRNN model for this system. Six small molecules with scoring rankings superior to the positive compounds were selected for molecular dynamics simulations. RMSD results show that IDH305, M1 and M2 stabilized after 200 ns and RMSD fluctuations remained within a range of 2 Å. IDH305 fluctuated between 275 ns and 400 ns but within a range of 2 Å. M3 and M6 RMSD after 350 ns fluctuations gradually stabilized. The results of the RMSF analysis suggest that there are notable variances in RMSF values (>2 Å) in regions 75–100, 125–200 and 275–300, which are mainly situated close to the binding site. Generally, increased flexibility is associated with decreased density and intramolecular hydrogen bonds, potentially affecting the distance between crucial residues. Rg, H-bond and SASA results indicate that all six small molecules have a higher affinity and more stable relationship with the target proteins during the simulation at 400 ns. Rg, H-bond and SASA results indicate that all six small molecules have higher affinity and more stable binding to the target protein during the simulation at 400 ns. The results of DCCM indicate that different substitutions at the same position can lead to variations in the internal dynamics of mDIH1. The lowest energy conformational relationship analyses of the six compounds revealed that six small molecules have a similar binding interaction pattern to IDH305, and the similar interaction pattern may be the reason for the higher affinity. Moreover, the binding modes of M1, M2, M3, M4, M5 and M6 with mIDH1 showed a higher degree of interaction with the active site compared to IDH305. This observation is consistent with the results obtained from IDH305. Observation is consistent with the results obtained from their binding affinity analyses. Binding free energy analyses of the six candidate molecules revealed that among these complexes, M1 had the best binding affinity and all the complexes showed a higher degree of interaction with the active site compared to the positive control IDH305 (−19.817 kcal/mol) with better binding affinity, in agreement with the molecular docking results.

Although the BRNN model-generated molecules showed superiority over the reported 625 mIDH1 inhibitors in terms of physicochemical properties such as synthesizability, drug-like properties and LogP, and that six molecules screened from the BRNN model-generated molecules were superior to the positive drug in terms of molecular docking, molecular dynamics simulations and binding free energy calculations, experimental validation is lacking. BRNN models are trained using fragments derived from real molecules, so the predicted molecules have a high degree of drug-like properties and synthesizability, but the use of reported molecular fragments for model training can result in generating molecules that are less novel, making it difficult to form compounds with entirely new structural types, and may not be able to bypass some patents. The effectiveness of BRNN models depends heavily on the quality and diversity of the training data. If the training data is not rich enough or biased, the generated results may also be limited or biased.

## 5 Conclusion

In this study, by comparing the molecules generated by the BRNN model with the molecules generated by DS scaffold hopping, it was found that the molecules generated by the BRNN model had better diversity, drug-likeness, docking score and synthesizability. Ten potential mIDH1 inhibitors were obtained through virtual screening the molecules generated by the BRNN model. The molecular dynamics simulation results showed that compound M1, M2, M3, M4, M5, M6 exhibited the best binding properties in all energy aspects, which have the potential to act as mIDH1 inhibitors. This study provides a drug design strategy by integrating deep learning, molecular docking and molecular dynamics simulation technology, provides new candidate drugs for the treatment of mIDH1-related tumors, and also provides a theoretical and practical basis for future drug design and development. Future work will include experimental verification of the biological activity of these compounds and further optimization of their structures to enhance their therapeutic effects as anticancer drugs.

## Data Availability

All relevant data from the study is included in the article. Further inquiries can be directed to the corresponding authors.
